# The Epidemic and the Public

**Published:** 1918-11-23

**Authors:** 


### The Hospital

The Workers' Newspaper of Administrative Medicine and Institutional Life, Administration, National Insurance and Health.

No. 1694, Vol. LXV. SATURDAY, NOVEMBER 23, 7918. PRICE TWOPENCE

### THE EPIDEMIC AND THE PUBLIC.

The recent and, unhappily, still prevailing epidemic of influenza has, of course, its scientific aspects, and with these the bacteriological and other experts are necessarily engaged—not, it would seem, so far, with very fruitful results. The difficulties are great, and a commanding success is certainly not within reach, and hardly, indeed, within sight. Neither a certain cure nor sure prevention is at hand. Here Science must be the helper, and she must be trusted to do her best. So far as public opinion may aid the task, what is needed is encouragement, not ignorant censure. The latter is open to all the critics, but it does not help the situation. In medicine, as we are now told is the case in politics, it is the criticism of the expert, not of the partisan, that is of constructive value. For our own part, we have every confidence that both the physician and the laboratory worker are doing their utmost both to help the individual sufferer and to save the community from the repetition of a like disaster. A triumph over all the ills to which flesh is heir is a highly improbable development. But some mitigation may be anticipated, and we trust that this will include the liability to such outbreaks as the one that has recently made desolate so many homes.

There are certain broad and general relations of the epidemic that are of immediate public interest. In districts not a few it is certain that the medical services have been quite inadequate to afford even a minimum of assistance to the victims. The demands of the Army have depleted the ranks of the doctors, and as a result the civil population has suffered. Such a possibility was pointed out long ago, and, so far as organised civilian effort was possible, attempts were made to moderate the official claims. We are not going to condemn these claims, but those who made them must accept the full responsibility, and the warning given has received abundant justification. The experience emphasises the need for continued pressure on the War Office in order to obtain as early and as large a return as possible of medical practitioners to their usual civilian practices. No doubt the immediate duty of the War Office is to see that due medical provision is made for the wants of the soldiers, but the authorities must be made to contemplate the situation as it actually exists and must be forced to realise that they cannot free themselves from the claims of the civilian public. The position is now entirely changed. The medical provision necessary for the Army can be exactly calculated, and no provision has to be made for the sudden, crowded and uncertain casualties of the battlefield. Hence the problem is one of simple and confident estimate, and the public has a right to expect that this shall be framed and adopted without delay.

Arising out of the suffering consequent on the short supply of doctors is an illustration of the importance to the public of the general practitioner, and of the need of ready and prompt access to him. The present is probably the first occasion during this generation in which there has been considerable and even widespread suffering because medical help could not be obtained, or could be obtained only in a hurried and incomplete fashion. It has been simply impossible for many practitioners to give anything like adequate attention to the numerous patients crowded on their hands. The lesson is an obvious one. There is a large public interest concerned in the number of doctors available for family practice, and whatever influence reduces this number below a certain level has unhappy consequences for the sick and suffering. Just now, schemes of medical reform abound. It is for the guardians of the general welfare to see that no scheme is adopted that deprives the community of ready medical service. Once rob general practice of its attractions and of its opportunities and the result just indicated must necessarily follow. To read some comments it might appear that all that is medically necessary for the public is an army of "specialists." The ideal is an ill-informed one. The supply of ready succour in times of emergency and the carrying of the help of medicine into the homes of the people depend on the family practitioner, and it will be an ill day for the community when this governing principle is forgotten. The claim for his preservation and advancement rests on the broadest of motives. namely, the protection of the public interest.

Another general demonstration exhibited during the epidemic has been the inadequacy in certain respects of our hospital system. Applications for admission to the hospitals have poured in on the authorities, and only a limited proportion of them could be granted. There has, so at least it seems to us, been some want of flexibility in this matter. The position was an urgent and acute one, and apart from the hospitals, it was certain that many of the sufferers could receive neither medical care nor nursing assistance. To meet such a situation more available beds should have been secured by the exclusion, and even by the evacuation, of patients suffering from non-urgent illnesses, and this proposition would include a number of surgical ailments. Some endeavour in this direction was, we are aware, attempted, but there was nothing like a general recognition of the necessity. Further, we must again remark that owing to the inaction of the War Office there are still numerous beds in the civilian hospitals occupied by convalescent soldiers. The soldiers merit whatever can be done for them, but this is no reason for allowing the War Office to meet an official responsibility at the cost of civilian sufferers. Such a state of matters has long been without justification, and the recent epidemic has aggravated the mischief.

